# Clinical Significance of HLA-DQ Antibodies in the Development of Chronic Antibody-Mediated Rejection and Allograft Failure in Kidney Transplant Recipients

**DOI:** 10.1097/MD.0000000000003094

**Published:** 2016-03-18

**Authors:** Hyeyoung Lee, Ji Won Min, Ji-Il Kim, In-Sung Moon, Ki-Hyun Park, Chul Woo Yang, Byung Ha Chung, Eun-Jee Oh

**Affiliations:** From the Department of Laboratory Medicine (HL, E-JO); Division of Nephrology, Department of Internal Medicine (JWM, CWY, BHC); Department of Surgery, Seoul St. Mary's Hospital, College of Medicine (J-IK, I-SM); and Department of Biomedical Science (K-HP), Graduate School, Catholic University of Korea, Seoul, Republic of Korea.

## Abstract

With the development of the single antigen beads assay, the role of donor specific alloantibody (DSA) against human leukocyte antigens in kidney transplantation (KT) has been highlighted. This study aimed to investigate the clinical significance of DQ-DSA detected at renal allograft biopsy. We evaluated 263 KT recipients who underwent allograft biopsy and DSA detection at the same time. Among them, 155 patients who were nonsensitized before transplantation were selected to investigate the role of de-novo DQ-DSA. Both the total and nonsensitized subgroup was categorized into 4 groups each according to DSA results as: DQ only, DQ + non-DQ, non-DQ, and no DSA. In the total patient group, post-KT DSA was positive in 79 (30.0%) patients and DQ-DSA was most prevalent (64.6%). In the nonsensitized subgroup, de-novo DSAs were detected in 45 (29.0%) patients and DQ-DSA was also most prevalent (73.3%). The DQ only group showed a significantly longer post-KT duration compared to the other groups (*P* < 0.05). The overall incidence of antibody-mediated rejection (AMR) was 17.9%. B-DSA, DR-DSA, and DQ-DSA were associated with AMR (*P* < 0.05), but in the analysis for chronic AMR, only DQ-DSA showed significance in both the total and the nonsensitized subgroup (*P* < 0.05). On comparison of Banff scores among groups, those representing humoral immunity were significantly dominant in all DSA positive groups compared to the no DSA group (*P* < 0.05), and higher scores of markers representing chronic tissue injury were more frequently detected in the groups with DQ-DSA. The worst postbiopsy survival was seen in the DQ + non-DQ group of the total patient group, and patients with de-novo DQ-DSA showed poorer graft survival in the nonsensitized subgroup compared to the no DSA group (*P* < 0.05). In the multivariate analysis, de-novo DQ-DSA was the only significant risk factor associated with late allograft failure (*P* < 0.05). Our study is the first to demonstrate the association of DQ-DSA with detailed histological findings representing chronic AMR. These findings suggest that the detection of DQ-DSA in nonsensitized patients is significantly associated with the development of chronic AMR and late allograft failure. Therefore monitoring of DQ-DSA not only in sensitized patients, but also nonsensitized patients may be necessary to improve long-term allograft outcomes.

## Abstract 2

Supplemental Digital Content is available in the text

## INTRODUCTION

Many previous reports showed that detection of donor specific alloantibody (DSA) against human leukocyte antigens (HLA) is significantly associated with chronic antibody-mediated allograft tissue injury, which results in poor allograft outcome.^1,2^ Among various types of donor specific HLA antibodies according to HLA locus, the role of DQ-DSA in transplantation has not yet been widely reported as DQ-alpha and DQ-beta are relatively newer testable antigens that have only recently been considered for routine testing.

A few years ago, single antigen bead assay with Luminex technology was introduced for the detection of DSA in kidney transplantation (KT), and it enabled accurate detection and research in regard to the clinical role of DQ-DSA in KTs.^3–11^ Several previous studies showed that DQ-DSA is frequently developed in a de-novo pattern, which is significantly associated with unfavorable allograft outcomes.^9–11^ Another report showed that DQ-DSA exhibited more resistance to antirejection treatment, and therefore emphasized the importance of early detection.^8^ However, the significance of posttransplant DQ-DSA detection, especially de-novo DQ-DSA, in the prediction of chronic allograft rejection and long-term graft survival in comparison with other types of DSA, has been sporadically reported and has not been fully investigated.

In this regard, we analyzed the results of DSA detection, especially focusing on DQ-DSA in KT recipients who underwent allograft biopsy in this study. We investigated the association between DQ-DSA detection and specific allograft biopsy findings and also evaluated the impact of DQ-DSA on long-term allograft outcome. In addition, we did a subgroup analysis in nonsensitized pretransplant patients to define the impact of de-novo DQ-DSA.

## MATERIALS AND METHODS

### Patients

**FIGURE 1:**
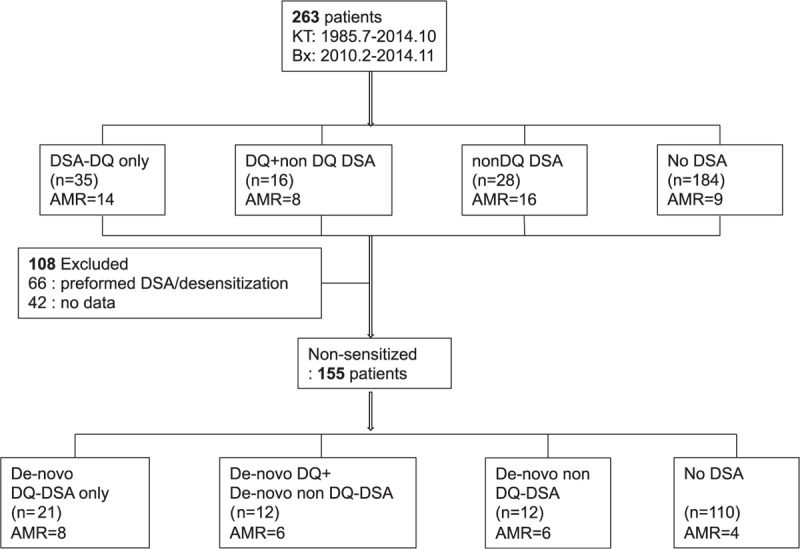
Flowchart of patients included and excluded from the study. AMR = antibody-mediated rejection, Bx = biopsy, DSA = donor specific alloantibody, HLA = human leukocyte antigens, KT = kidney transplantation.

We included 263 kidney-transplant recipients in whom we performed allograft biopsy under the suspicion of rejection or according to protocol, between February 2010 and November 2014 at Seoul St. Mary's hospital. The demographics of these patients are shown in Table 1. Out of 263 patients, 66 patients showed preformed DSA and/or required desensitization therapies. In another 42 patients, information for preformed DSAs was not available. Therefore, further analysis for de-novo DSA was done in the remaining 155 patients (Figure 1). For the detection of posttransplant DSA, serum sampling was performed within a day of the allograft biopsy. We divided the total patients group and the nonsensitized subgroup into 4 groups each, according to DSA results at the time of allograft biopsy into DQ only (those with only DQ-DSA), DQ + non-DQ (those with DQ-DSA, as well as DSA against HLA-A, B, DR), non-DQ only (those with DSA directed against an HLA-A, B, and DR, but without DQ-DSA), and no DSA (those without any detectable DSA) groups. We compared the pretransplant clinical characteristics, allograft biopsy findings and clinical outcomes after allograft biopsy among the DSA groups in the total patients group and in the nonsensitized subgroup. Allograft function was determined by calculating the estimated glomerular filtration rate (GFR) using the Modification of Diet in Renal Disease formula (GFR = 186.3 × serum creatinine^−1.154^ × age^−0.263^ × 0.742 if female).^12^ Amount of proteinuria was determined by calculating the protein/creatinine (P/C) ratio in the spot urine chemistry test. This study was approved by the institutional review board of Seoul St. Mary's Hospital. A written informed consent form for HLA typing test was signed by all patients.

**TABLE 1:**
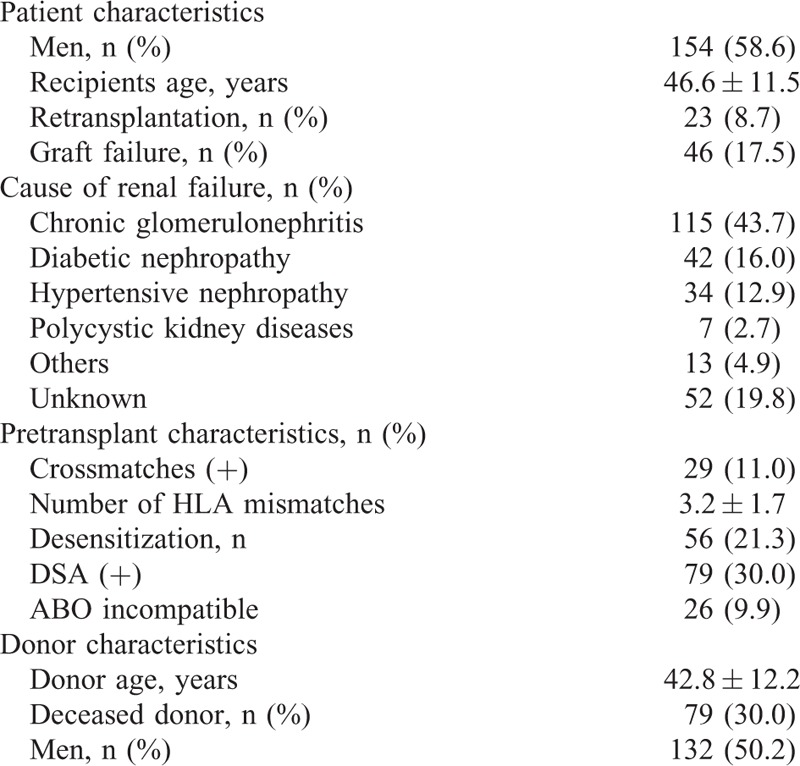
Demographic Parameters of the Total Study Population (n = 263)

### Induction and Maintenance Immune Suppressant Regimen

The typical immunosuppressive regimen at our center has previously been described.^13,14^ In brief, tacrolimus or cyclosporine was administered in combination with mycophenolate mofetil and prednisolone. Basiliximab was administered as induction therapy. In patients with DSA, only tacrolimus was administered as the main regimen.

### HLA Typing, HLA Antibodies, and DSA

HLA-A, B, DR, DQB1 typing was performed by the DNA molecular typing method using sequence-specific oligonucleotide probes with LIFECODES HLA SSO typing kits (Immucor, Stamford, CT). Lifecodes LSA Class I and Class II kits (Gen-Probe Transplant Diagnostic Inc., Stamford, CT) or LABScreen Single Antigen (One Lambda Inc., A Thermo Fisher Scientific Brand, Canoga Park, CA) were used for detecting HLA antibodies in recipient sera. According to the manufacturer's instructions, 10 μL of each serum sample were used and the fluorescence intensities of the samples were measured by using a Luminex 200 system (Luminex Corp., Austin, TX). Beads with median fluorescence intensity (MFI) of greater than 1000 were defined as positive. MFI of HLA-Abs were classified as weak (1000–5000), moderate (5000–10,000), and strong (>10,000) groups. A positive de-novo DSA was defined as a new antibody not present in pre-KT serum with donor specificity.

### Diagnosis and Treatment of Allograft Rejection

Allograft tissue biopsy findings were diagnosed according to the revised Banff 2007 classification.^15^ Indirect immunofluorescence staining was performed using monoclonal antibodies against complement protein C4d (Biogenesis, Poole, UK; dilution1:50) for detecting C4d deposition. C4d positivity was defined as diffuse (>50%) and linear staining of peritubular capillaries. When T cell mediated rejection was detected, we used 3 to 5 daily boluses of intravenous methylprednisolone (250 mg/day), followed by a 5 to 7 day oral steroid taper. When T cell mediated rejection proved resistant to steroid therapy, antithymocyte globulin was applied within 5 days of treatment. When antibody-mediated rejection (AMR) was diagnosed, we used plasmapheresis with intravenous immunoglobulin (IVIg) and rituximab in patients who did not responded to plasmapheresis/IVIg therapy. In chronic AMR, patients were treated with rituximab, IVIg for 4 days, and twice daily boluses of intravenous methylprednisolone (250 mg per bolus) for 3 days, followed by a 5 to 7 day oral steroid taper.

### Statistical Analysis

Statistical analyses were performed with the statistical package MedCalc version 15.5 (MedCalc, Mariakerke, Belgium). Comparisons of categorical variables between 2 groups were performed by Chi-square and Fisher exact tests. Multivariable logistic regression analysis and Cox regression multivariable analysis were applied to confirm the association on graft outcome. Graft survival rates were computed according to the Kaplan–Meier survival analysis. All reported *P* values are 2-sided, and *P* value <0.05 was considered statistically significant.

## RESULTS

### Posttransplant Detection of HLA-DSA

**FIGURE 2:**
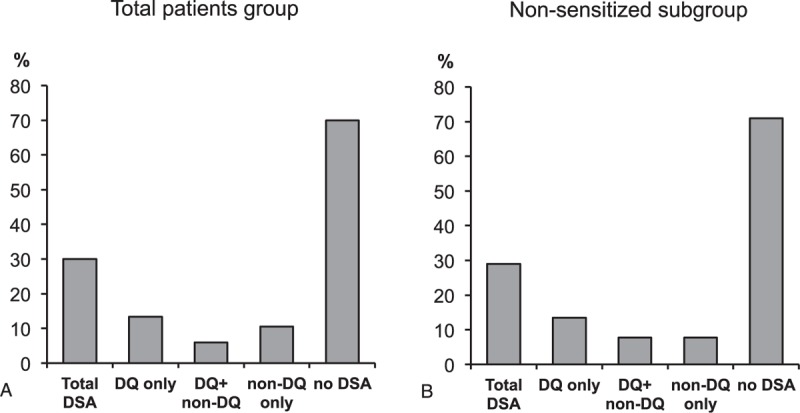
Prevalence and distribution of HLA-DSA according to groups. (A) Distribution of total patients according to the presence of DQ and non-DQ DSA. (B) Distribution of the nonsensitized subgroup patients according to the presence of DQ and non-DQ DSA. DSA = donor specific alloantibody, HLA = human leukocyte antigens.

Post-KT DSAs were positive in 79 patients (30.0%). Of them, 51 patients (64.6%) developed DQ-DSA and the most prevalent DQ-DSA were DQ6 (33.3%), DQ7 (23.5%), and DQ2 (23.5%). Of 51 patients with DQ-DSA, 35 (68.6%) developed only DQ-DSA (DQ only group), whereas 16 (31.4%) developed DQ-DSA along with DSA of other specificities (DQ + non-DQ group). DQ-DSA was most frequently accompanied by DSA against HLA-DR (13 patients, 81.3%), followed by 5 patients (31.3%) with DSA against HLA-A, and 2 patients (12.5%) with DSA against HLA-B. The remaining 28 patients with non-DQ-DSA were placed in the non-DQ-DSA group. In the non-DQ-DSA group, DSA against DR was most prevalent (14 patients, 50.0%), followed by HLA-B (12 patients, 42.9%), HLA-A (7 patients, 25.0%). In the subgroup analysis for de-novo DSA, 45 out of 155 patients (29.0%) showed positive post-KT DSA. The most prevalent DSA was DQ-DSA (33/45 [73.3%]) and the most common locus of de-novo DQ-DSA were DQ6 (13 patients, 39.4%), DQ2 (9 patients, 27.3%), and DQ7 (8 patients, 24.3%). Twenty-one patients showed isolated DQ-DSA, and 12 patients had both DQ-DSA and non-DQ DSA. Of the 12 patients, 10 patients showed DSA against DR, 2 with DSA-B, and 2 had DSA-A. The remaining 12 patients in the non-DQ-DSA groups showed that 7 patients had DSA against HLA-B, 5 had DSA against HLA-DR, and 3 had DSA against HLA-A (Figure 2).

### Comparison of Baseline Clinical Characteristics According to DSA Group

In total patients group, there were no significant differences detected between clinical characteristics such as age, gender, primary renal disease, percentage of ABO incompatible KTs, donor type, and proportion of patients desensitized (Table 2). Posttransplant duration was significantly longer in the DQ only group compared to the other groups (*P* < 0.05). Proportion of retransplant patients was significantly higher in the DQ only and non-DQ groups compared to the no DSA group (*P* < 0.05). In the subgroup analysis for de-novo DSA in nonsensitized patients (n = 155), posttransplant duration was also longer in the DQ only and DQ + non-DQ groups compared to the no DSA group.

**TABLE 2:**
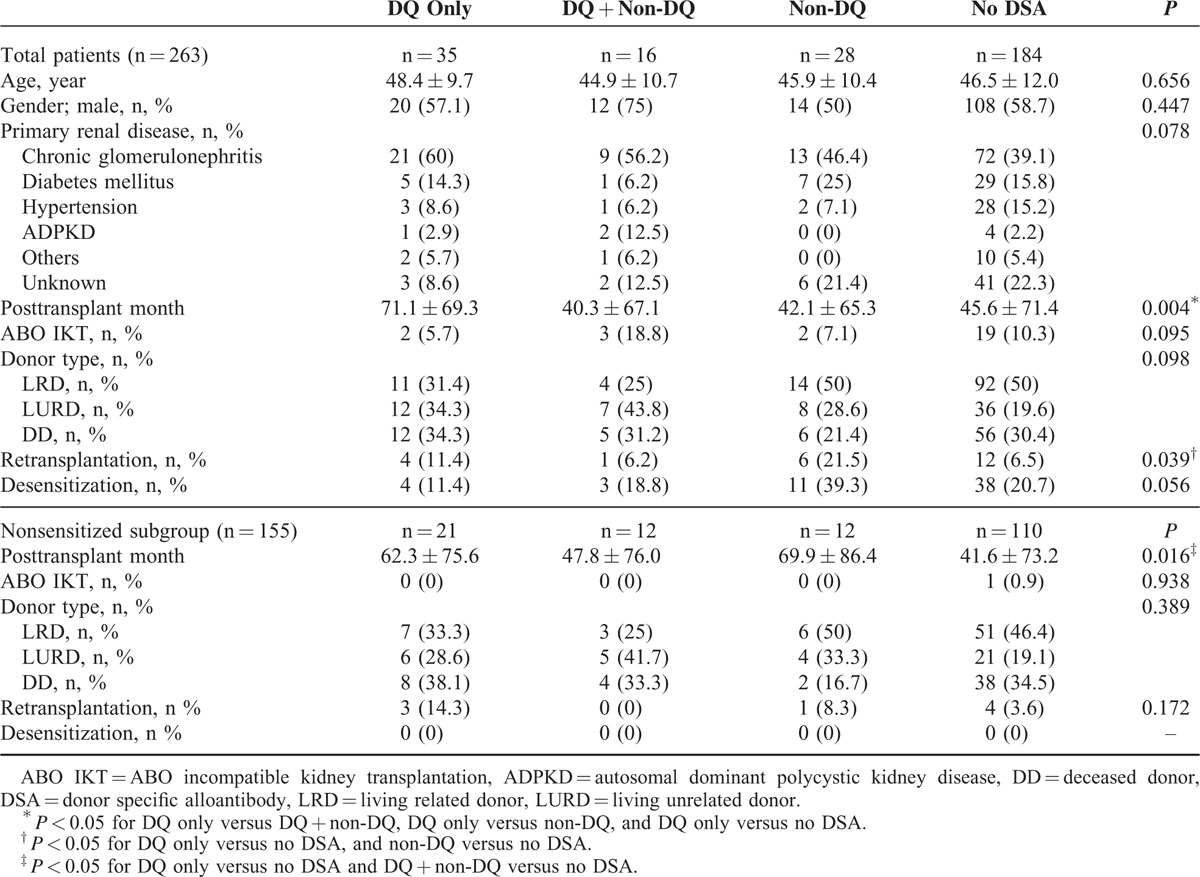
Comparison of Clinical Characteristics Between DQ only, DQ + Non-DQ, Non-DQ, and No DSA Group

### Comparison of Biopsy Findings According to DSA Group

We analyzed the biopsy results in the 4 groups according to DSA results in the total and nonsensitized subgroup (Table 3). The overall incidence of AMR was 17.9% in total patients, and the incidence of total AMR was higher in the DQ only, DQ + non-DQ, non-DQ compared with the no-DSA groups, and DQ only group also had similar incidence of AMR compared to the DQ + non-DQ or non-DQ groups (DQ only: 40.0%, DQ + non-DQ: 50%, non-DQ: 57.1%). Unlike other DSA groups, the DQ only group with AMR showed higher frequency of chronic AMR (10/14 patients) compared to acute AMR (4/14) (*P* < 0.05). On univariate analysis, all A, B, DR, or DQ-DSAs were associated with AMR (Table 4) and on multivariate analysis, B-DSA (odds ratio [95% confidence interval], 11.01 [2.96–41.03]; *P* = 0.0004), DR-DSA (5.77 [2.18–15.31]; *P* = 0.0004), and DQ-DSA (5.34 [2.43–11.76]; *P* < 0.0001) were associated AMR. But, in the analysis for chronic AMR, only DQ-DSA (7.56 [2.76–20.66]; *P* = 0.0001) showed significance.

**TABLE 3:**
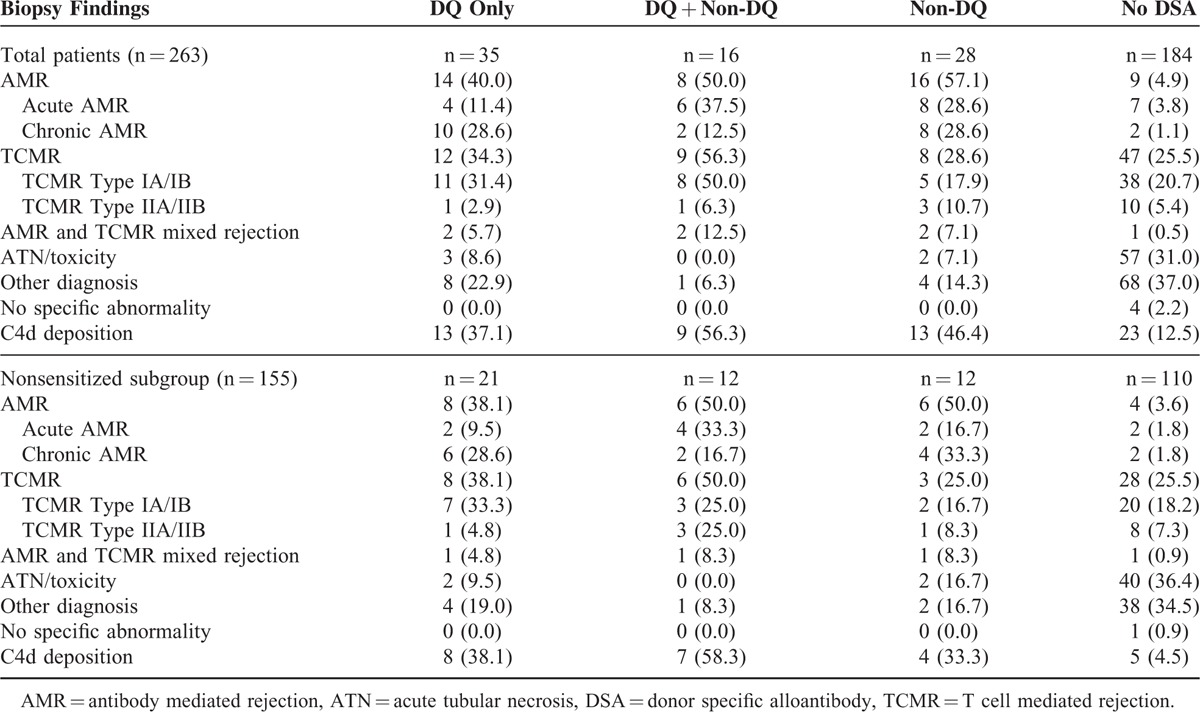
Biopsy and Clinical Findings According to DSA Group

**TABLE 4:**
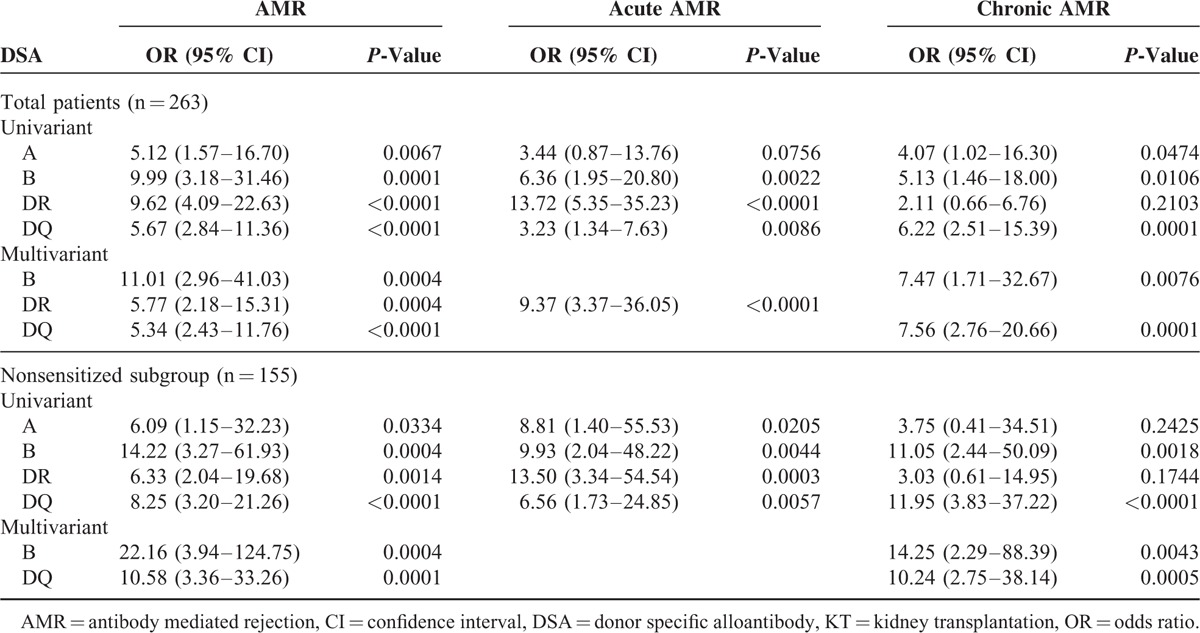
Analysis of Association of Post-KT DSA and AMR

In the nonsensitized subgroup analysis, the overall incidence of AMR was 15.5% and it was higher in the de-novo DQ only, DQ + non-DQ, non-DQ groups compared to the no-DSA groups as in the total patient group. On multivariate analysis, de-novo B-DSA (22.16 [3.94–124.75]; *P* = 0.0004) and de-novo DQ-DSA (10.58 [3.36–33.26]; *P* = 0.0001) were associated with AMR. However, for chronic AMR, de-novo DQ-DSA (10.24 [2.75–38.14], *P* = 0.0005) alone showed significance association. The strength of DSA did not show significant association with the incidence of AMR (weak MFI: 35.7%, moderate MFI: 21.4%, and strong MFI: 42.9%) in both the total patient group and nonsensitized subgroup.

### Comparison of Banff Score and Microvascular Inflammation Score Distribution Associated With Humoral Immunity

**FIGURE 3:**
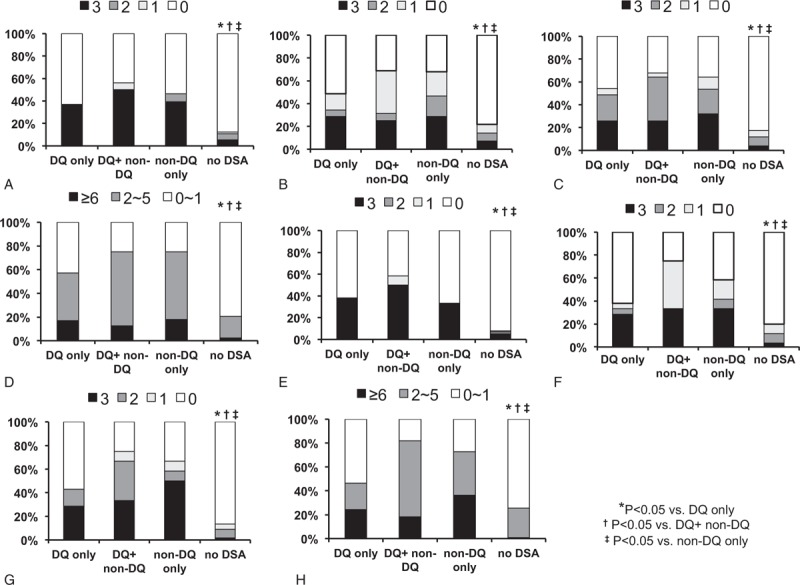
Comparison of Banff score and microinflammation score distribution associated with humoral immunity. In the total patients (A) C4d score, (B) g score, (C) ptc score, and (D) MVI scores were more frequently distributed as high score in other groups compared to the no DSA. Similarly (E) C4d score, (F) g score, **(**G) ptc score, and (H) MVI scores of the nonsensitized subgroup showed larger distribution of higher scores in the other groups compared to the no DSA group. ^∗^*P* < 0.05 versus DQ only group; ^†^*P* < 0.05 versus DQ+ non-DQ group; ^‡^*P* < 0.05 versus non-DQ only group. g = glomerulitis, MVI = microvascular inflammation, ptc = peritubular capillaritis.

The distribution of specific histologic findings based on Banff scores, especially focusing on scores associated with antibody-mediated tissue injury, was compared among each group (Figure 3). Higher scores of histologic markers representing humoral immunity, such as the C4d, glomerulitis (g), and peritubular capillaritis (ptc) scores, were significantly dominant in the DQ only, DQ + non-DQ, and non-DQ only groups compared to the no DSA group (*P* < 0.05, Figure 3A–C). And, the DQ only group showed similar histologic findings compared to the non-DQ group (*P* > 0.05). The microvascular inflammation (MVI) score, calculated by adding the g and ptc scores, was also found to be significantly higher in the DQ only, DQ + non-DQ, and non-DQ only groups compared to the no DSA group (*P* < 0.05, Figure 3D). Similarly, in the analysis of the nonsensitized subgroup, C4d, g, ptc, and MVI scores were all significantly dominant in the DQ only, DQ + non-DQ, and non-DQ only groups compared to the no DSA group (*P* < 0.05, Figure 3E–H), and the DQ only group showed similar scores compared to the non-DQ group (*P* > 0.05).

### Comparison of Banff Score Distribution Associated With Chronic Changes

**FIGURE 4:**
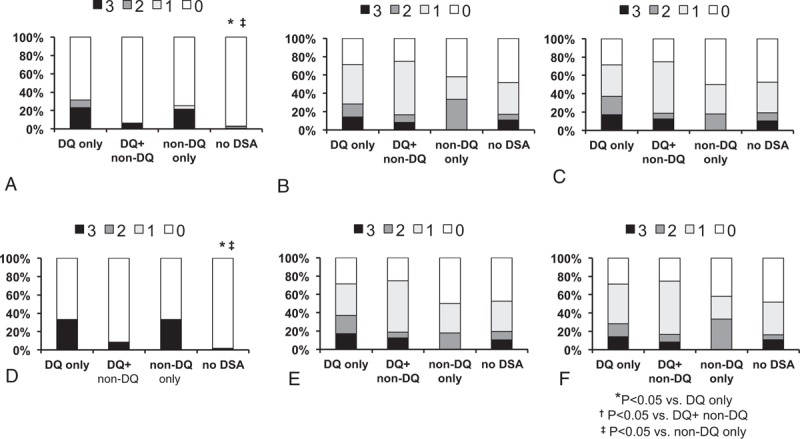
Comparison of Banff score distribution associated with chronic changes. In the total patient group (A) higher cg scores were more frequently distributed in the DQ only and non-DQ only groups, while (B) ct score and (C) ci showed no significant difference in score distribution among the groups. In the nonsensitized subgroup also, higher (D) cg scores were more frequently distributed in the DQ only and non-DQ only groups, while (E) ct score and (F) ci showed no significant difference in score distribution among the groups. ^∗^*P* < 0.05 versus DQ only group; ^†^*P* < 0.05 versus DQ+ non-DQ group, ^‡^*P* < 0.05 versus non-DQ only group. cg = glomerulosclerosis, ci = interstitial fibrosis, ct = tubular atrophy.

We also compared Banff scores associated with chronic changes in the allograft tissue. Glomerular sclerosis (cg) score was significantly higher in the DQ only and non-DQ only groups compared to the DQ + non-DQ group and no DSA group (*P* < 0.05, Figure 4A). Tubular atrophy (ct) and interstitial fibrosis (ci) scores showed increasing tendency in the DQ only, DQ + non-DQ groups compared to non-DQ only and no DSA groups, but did not show statistical significance (*P* = 0.095 and 0.083, respectively, Figure 4B, C). Results in the nonsensitized subgroup analysis were again similar to the total patient group. The cg score was significantly higher in the DQ only and non-DQ only groups compared to the no DSA group (*P* < 0.05, Figure 4D), and although ct and ci scores also showed higher score distribution in the DQ only group compared to the other groups, results were not statistically significant (*P* = 0.207 and 0.154, respectively, Figure 4E, F).

### Comparison of the Impact of DSA on Postbiopsy Allograft Survival

**FIGURE 5:**
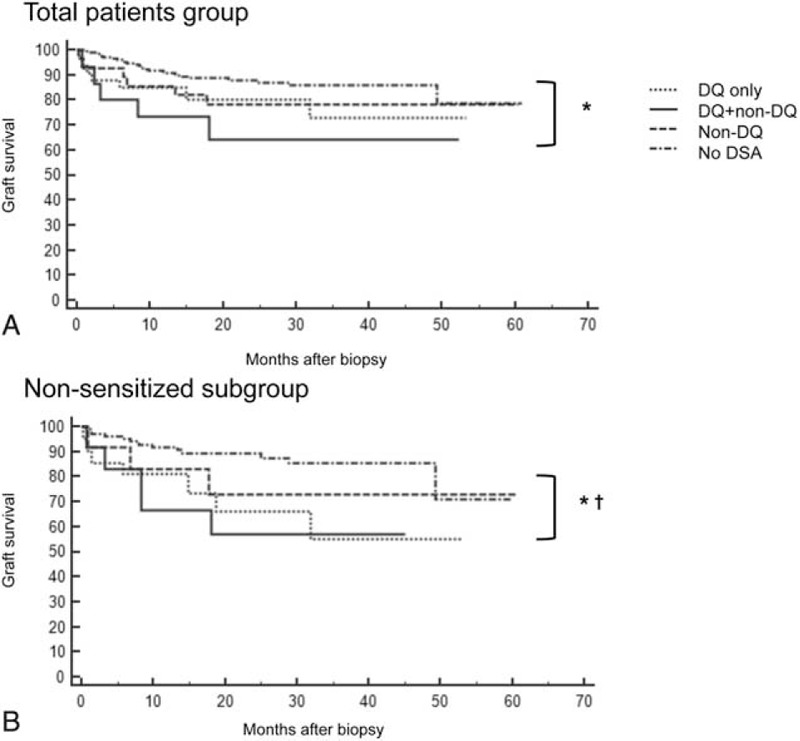
Postbiopsy graft survival of patients. In the total patients group (A), the DQ + non-DQ group showed significantly lower postbiopsy survival compared to the no donor specific alloantibody (DSA) group. In the nonsensitized subgroup (B), the DQ + non-DQ as well as the DQ only group showed significantly lower postbiopsy survival compared to the no DSA group. ^∗^*P* < 0.05 DQ+ non-DQ group versus no DSA group; ^†^*P* < 0.05 DQ only versus no DSA group.

The postbiopsy graft survival of the entire cohort according to DSA groups is shown in Figure 5A. The DQ + non-DQ group showed the worst postbiopsy survival with statistical significance compared to the no DSA group (*P* = 0.0026), but no significant difference compared to the other DSA groups. The postbiopsy graft survival in the nonsensitized subgroup is illustrated in the Figure 5B. Patients with de-novo DQ-DSA showed poor graft survival compared with no DSA (DQ only vs no DSA [*P* = 0.0039] and DQ + non-DQ vs no DSA [*P* = 0.0126]). Cox regression multivariable analysis was done to observe the impact of DSA on posttransplant allograft survival in the total patients group and in the nonsensitized subgroup (Table 5). In the total patients group, there were no significant risk factors significantly associated with allograft failure. But in the nonsensitized subgroup, detection of DQ-DSA was the only significant risk factor associated with allograft failure (hazard ratio 9.05 [3.21–25.51], *P* < 0.0001).

**TABLE 5:**
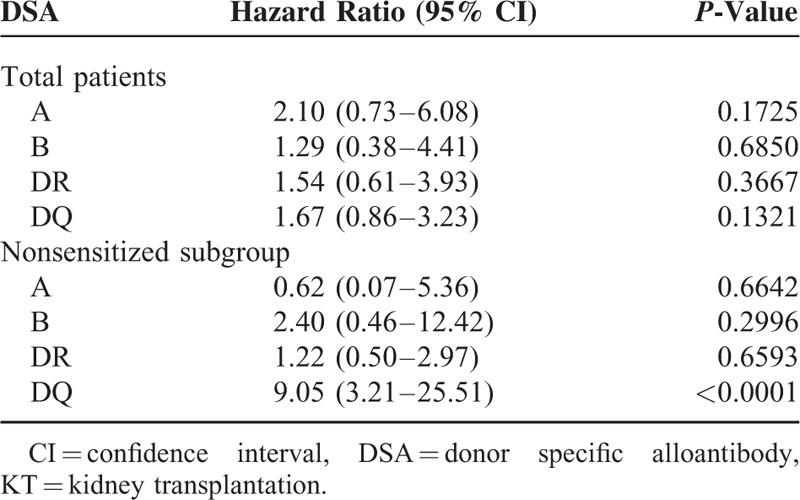
Cox Regression Multivariable Analysis for Post-KT DSA to Influence Allograft Failure

## DISCUSSION

Although increasing evidence highlights the detrimental effects of DSA in KT, researches focusing on the impact of DQ-DSA are still rare. In this study, we analyzed the result of DSA detection at the time of allograft biopsy and showed that DQ-DSA is the most commonly detected DSA type and furthermore it is associated with more frequent development of chronic AMR, which resulted in poor allograft outcome after allograft biopsy.

First, we investigated the frequency of DQ-DSA at the time of allograft biopsy and did a subgroup analysis of pretransplant nonsensitized patients for de-novo DQ-DSA. As a result, DQ-DSA proved to be the most commonly detected type not only in the entire cohort but also in the subgroup comprised of previously nonsensitized patients, which is consistent with previous studies.^9–11,16^ The high frequency of DQ-DSA found after transplantation may be explained by the high polymorphism of the genes which encode the DQ molecule (DQA1 and DQB1). Therefore, the immune system stimulated by sensitizing events such as transplantation, pregnancy, or transfusion is most likely to form antibodies against HLA-DQ.^16^ DQ-DSA was most commonly accompanied by DSA against HLA-DR in our study, which is also consistent with results from previous studies.^3,9,10^ This may be explained by the strong linkage disequilibrium between the DQ and DR locus, and which is probably why the two are often inherited together within the same racial or ethnic group.^17^

Second, we intended to investigate the clinical characteristics of patients with DQ-DSA compared to other patients. We found that posttransplant duration was significantly longer in the DQ only group compared to the other groups. This finding was also persistent in the subgroup analysis for de-novo DSA in pretransplant nonsensitized patients. In the analysis of the association between post-KT periods and de-novo DSA detection, less than 1 year, 1 to 5 years, more than 5 years, the proportion of de-novo DQ-DSA showed increasing tendency with post-KT periods even though no statistical significances was found (Supplementary Figure 1). These results may result from the characteristics of DQ-DSA. One previous study showed that HLA-DQ is not expressed in a normal kidney and only found when upregulated by an inflammatory process in the renal microvascular environment.^18^ Therefore, it is possible that formation of DQ-DSA may take a longer time compared to other types of DSA that are constantly expressed in renal tissue. Also, another explanation for the later detection of DQ-DSA could be that such antibodies are associated with a longer period of time before manifestation of renal dysfunction leading to attaining a cause for kidney biopsy.

Third, we investigated the association between DQ-DSA and allograft biopsy diagnosis. We found DQ-DSA to be associated with AMR, and its significance was more dominant in chronic AMR compared to other types of DSA, such as DR-DSA, which has a significant role in acute AMR. Indeed, chronic AMR showed increasing tendency in the DQ only group compared to other DSA groups. In addition, DQ-DSA of both the total group and the nonsensitized subgroup presented as significant risk factors for chronic AMR in the multivariate analysis. These findings are consistent with previous studies reporting the association of DQ-DSA with AMR.^10^ The reason for the dominant association of HLA-DQ to chronic AMR rather than acute AMR is unclear. But as we presented above, expression of HLA-DQ in the normal kidney is scanty in contrast to HLA-DR, which showed constituent expression, and usually showed upregulation during inflammatory process such as allograft rejection.^18^ Another study showed that specific types of HLA-DQ do not induce acute humoral immune reaction.^19^ Those differences of HLA-DQ and HLA-DR may result in the different immune action to allograft between DQ-DSA and DR-DSA. However, further investigation may be required for a clear conclusion about this issue.

Next, we investigated the association between DQ-DSA and specific allograft biopsy findings, especially those associated with humoral immunity and chronic changes. Previous studies in regard to the association between DQ-DSA and acute rejection did not examine specific biopsy findings.^9,10^ In this study, Banff scores such as C4d, g, and ptc scores were significantly higher in the groups with HLA-DSA (including both DQ and non-DQ) compared to the no DSA group. MVI score was also higher in the DSA positive groups. This is consistent with previous studies that reported the association of C4d and MVI scores with the presence of DSA and de-novo DSA.^20,21^ Of the Banff scores associated with chronic changes, the cg score was significantly higher in the DQ only and non-DQ only groups both in the total patients group and nonsensitized subgroup analysis. Those results are fully consistent with the higher prevalence of chronic AMR rather than acute AMR in patients with DQ-DSA. There have been a few previous studies that demonstrated the association of class II DSA, and more specifically DQ-DSA, with late AMR.^8,21^ To our knowledge however, our study is the 1st to demonstrate the association of DQ-DSA with more detailed histological findings representing humoral immunity and chronic changes.

Finally, we investigated the association of DQ-DSA on allograft outcomes after allograft biopsy. In both the total group and nonsensitized subgroup, patients showing DQ-DSA (DQ only and DQ + non-DQ groups) had significantly lower allograft survival rates compared to the no DSA group. Regarding the association of DQ-DSA with allograft survival after transplant surgery, only the DQ + non-DQ group showed significantly lower graft survival compared to the no DSA group in both the total group and nonsensitized subgroup (Supplementary Figure 2). However, multivariate analysis using Cox regression test showed that DQ-DSA, more specifically de-novo DQ-DSA, independently affects posttransplant allograft outcomes. As mentioned above, DQ-DSA was shown to be more strongly associated with chronic AMR, rather than acute AMR which in itself would have contributed to the development of late allograft failure.^22,23^ Indeed, we observed that posttreatment allograft function, represented by estimated GFR and amount of proteinuria, was significantly lower in the chronic AMR group even though it was similar in both acute and chronic AMR patients at the time of biopsy **(**Supplementary Figure 3). The poorer response to antirejection therapy can result in worse allograft outcome in chronic AMR patients compared to acute AMR group. Therefore, DQ-DSA which was more frequently detected in chronic AMR rather than acute AMR may show significantly poorer effects on allograft outcomes.

This study has its limitations. We did not perform serial monitoring for DSA, and therefore could not define the exact point in time in which DQ-DSA had developed and when and how this development would have affected allograft outcomes. Observation of serial DSA results by regular DSA monitoring in a larger cohort may further clarify this issue.

In conclusion, our results showed that detection of DQ-DSA is associated with the development of chronic tissue injury including chronic AMR in allograft biopsy findings, which resulted in increased risk of late allograft failure not only in total patients but also in the pretransplant nonsensitized subgroup analysis. Therefore, regular monitoring of DQ-DSA may be required to predict allograft outcomes and prevent DQ-DSA associated allograft tissue injury even in patients not sensitized before transplantation.

## Supplementary Material

Supplemental Digital Content
