# Supplementary material for: Clinical Significance of HLA-DQ Antibodies in the Development of Chronic Antibody-Mediated Rejection and Allograft Failure in Kidney Transplant Recipients
Source: Medicine (Baltimore). 2016 Mar 18;95(11):e3094. doi: 10.1097/MD.0000000000003094 (PMC4839928; doi:10.1097/MD.0000000000003094)

Supplementary figure 1. De-novo DSA detection according to post KT periods (less than year, 1 to 5 years, more than 5 years). The proportion of de-novo DQ-DSA showed increasing tendency with increase in post-KT duration, but no statistical significances was found.
Abbreviations; KT, kidney transplantation; DSA, donor specific HLA antibody

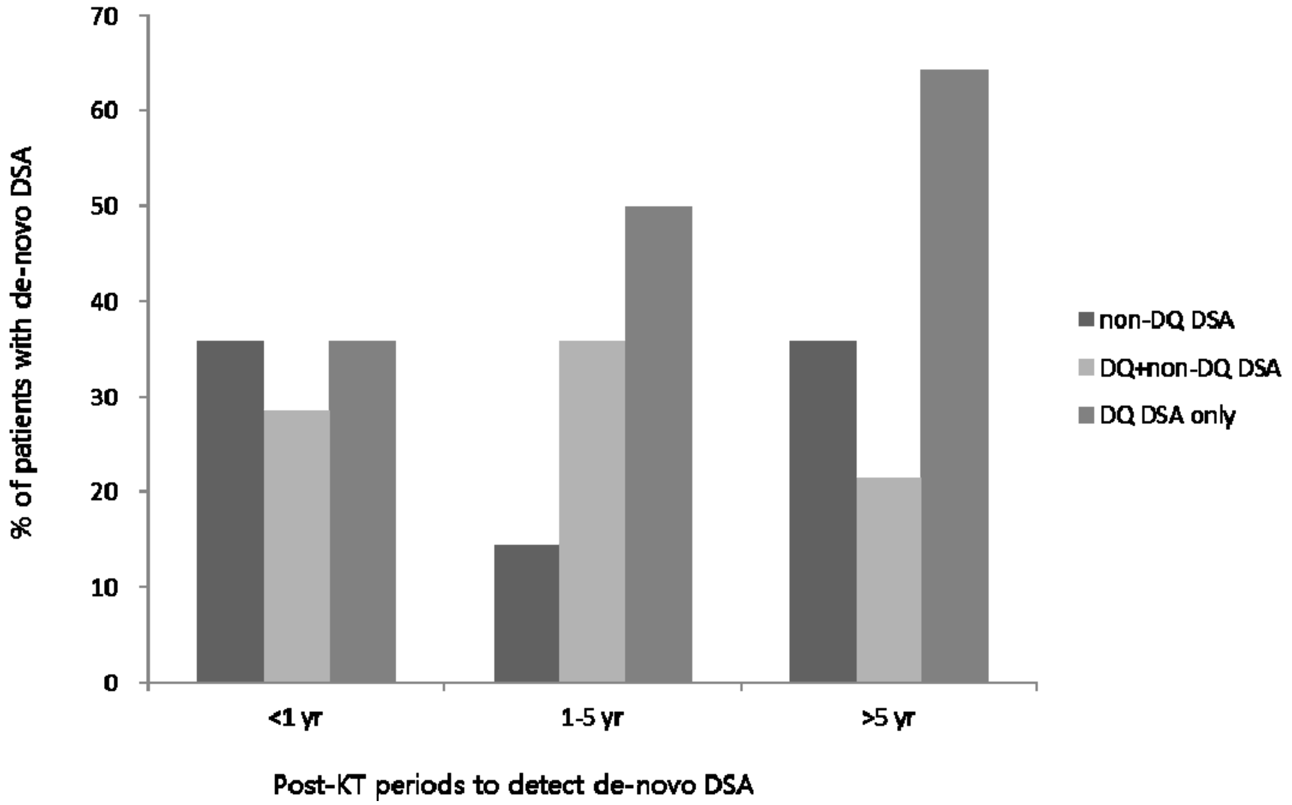

Supplementary figure2. Graft survival of total patients (A) and non-sensitized subgroup (B) after the transplant surgery. The DQ+non-DQ group showed significantly lower graft survival compared to the no DSA group (P=0.0026 in total patients, P=0.0064 in non-sensitized patients).

*P<0.05 DQ+ non-DQ group vs. no DSA group

(A) Total patients group

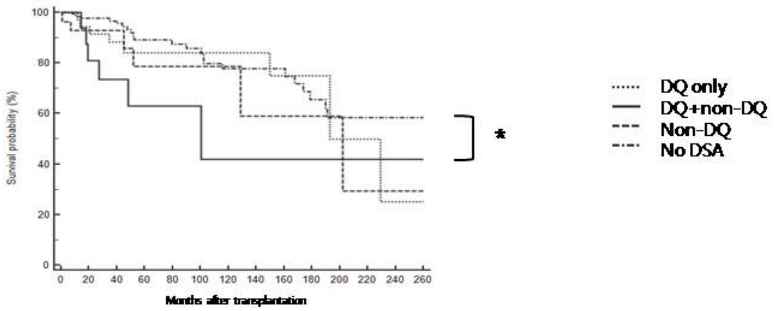

(B) Non-sensitized subgroup

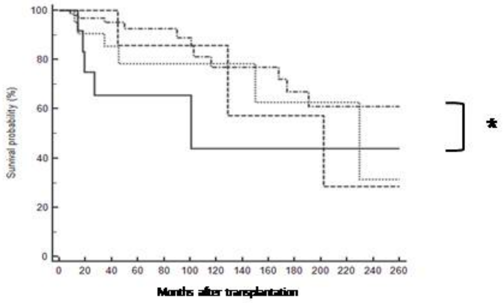

Supplementary figure3. Comparison of post-treatment renal function in acute and chronic AMR patients. Renal function demonstrated by (A) MDRD eGFR was significantly higher in acute AMR patients at 6 months after treatment and (B) amount of proteinuria was also significantly lower in acute AMR patients at 1, 3 and 6 months after treatment. Abbreviations; AMR, antibody mediated rejection; MDRD eGFR, estimated glomerular fitration rate using the Modification of Diet in Renal Disease; P/C ratio, protein/creatinine ratio

*P<0.05

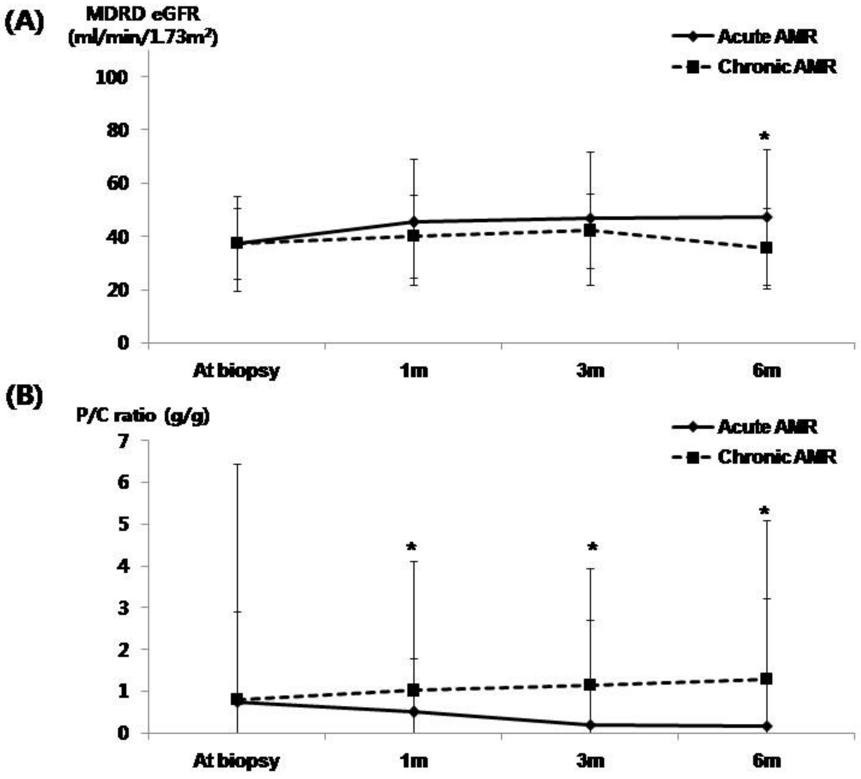

Supplement: Supplemental Digital Content [file medi-95-e3094-s001.pdf]
